# Adjuvant Approaches in Fully Resected Stage III and IV Cutaneous Melanoma: Where Are We Now?

**DOI:** 10.3390/cancers18121961

**Published:** 2026-06-16

**Authors:** Luisa Piccin, Valentina Guarneri, Michele Del Vecchio, Andrea Spagnoletti, Paolo Fava, Gabriele Roccuzzo, Carolina Cimminiello, Nikolaos Papadopoulos, Alessandro Minisini, Jacopo Costa, Jacopo Pigozzo

**Affiliations:** 1 Medical Oncology 2, Veneto Institute of Oncology IOV-IRCCS, 35128 Padova, Italy; luisa.piccin@iov.veneto.it (L.P.); valentina.guarneri@iov.veneto.it (V.G.); 2 Department of Surgery, Oncology and Gastroenterology, University of Padova, 35124 Padova, Italy; 3 Fondazione IRCCS Istituto Nazionale dei Tumori, 20133 Milano, Italy; michele.delvecchio@istitutotumori.mi.it (M.D.V.); andrea.spagnoletti@istitutotumori.mi.it (A.S.); 4 Department of Medical Sciences, University of Turin, 10126 Torino, Italy; paolo.fava@unito.it (P.F.); gabriele.roccuzzo@unito.it (G.R.); 5 European Institute of Oncology, IRCCS, 20141 Milano, Italy; carolina.cimminiello@ieo.it (C.C.); nikolaos.papadopoulos@ieo.it (N.P.); 6 Department of Oncology, Santa Maria della Misericordia University Hospital, Azienda Sanitaria Universitaria Friuli Centrale (ASUFC), 33100 Udine, Italy; alessandro.minisini@asufc.sanita.fvg.it (A.M.); costa.jacopo@spes.uniud.it (J.C.)

**Keywords:** melanoma, adjuvant therapy, immunotherapy, targeted therapy

## Abstract

Objectives: To describe the scientific evidence regarding adjuvant treatment for stage III and IV cutaneous melanoma and the unresolved issues in this setting. Methods: This review examines the main studies on adjuvant medical therapies approved over the years by the Food and Drug Administration and European Medicines Agency together with the main evidence related to the treatments referred to in the National Comprehensive Cancer Network and European Society of Clinical Oncology guidelines at the time of submission (May 2026) for stage III and IV cutaneous melanoma. A particular focus on immunotherapy (interferon, ipilimumab, and anti-PD-1 antibodies, both as monotherapy and in combination) and targeted therapy with anti-BRAF agents, either as monotherapy or in combination with MEK inhibitors, is given. Besides that, this work also evaluates the role of radiation therapy and addresses some unresolved issues, such as adjuvant therapy in stage IIIA and treatment selection in *BRAF*-mutated melanoma. Results: Adjuvant therapy for stage III and IV cutaneous melanoma has evolved over the years, starting with interferon and progressing to the use of immunocheckpoint inhibitors and targeted therapy. However, not all treatments that have proven effective in metastatic disease have subsequently played a role in the adjuvant setting. Conclusions: Currently, adjuvant treatment for stage III and IV cutaneous melanoma involves the use of anti-PD-1 antibodies (nivolumab and pembrolizumab) and dabrafenib plus trametinib if the patient has a *BRAF* V600 mutation. It was not possible to identify the adjuvant therapy of choice for BRAF-mutated melanoma, and several factors must be considered when deciding between immunotherapy and targeted therapy. The role of radiation therapy remains controversial and could be discussed by the multidisciplinary team as part of the adjuvant strategy in selected patients. Likewise, adjuvant therapy for stage IIIA melanoma should be carefully evaluated in light of the risk–benefit ratio.

## Simple Summary

Patients with stage III and stage IV melanoma are at high risk of recurrence after surgical resection and consequently dying from metastatic disease. It is now recognized that patients with resected stage III and stage IV melanoma may benefit from adjuvant systemic therapy after surgery, which aims to reduce the risk of disease relapse and potentially improve survival rates. Over the years, several treatment strategies have been studied in the adjuvant setting, leading to the current standard of care, which is based on the use of targeted therapy and immunotherapy. However, there are still unresolved issues regarding the adjuvant treatment of stage III and IV cutaneous melanoma, such as the role of radiation therapy, which treatment to recommend in the presence of a *BRAF* mutation, and whether to administer therapy in stage IIIA, which is characterized by a low risk of recurrence.

## 1. Introduction

A melanoma is a tumor composed of malignantly transformed melanocytes. These elements are neural crest-derived and melanin-secreting specialized cells, mostly located in the basal layer of the epidermis [1].

According to the seventh and eighth editions of the American Joint Committee on Cancer (AJCC) system, cutaneous melanoma stages III and IV include regional (nodal and/or satellite/in transit) and distant metastatic disease, respectively. Globally, these entities represent 7.5% and 2.4–4% of the more than 300,000 melanoma diagnoses performed in 2022 worldwide. These patients’ prognosis is very heterogeneous, ranging from 5-year melanoma-specific survival of 93% or 32% (for stage IIIA and stage IIIC, respectively) to a probability of death after one year of 67% (for extra-regional disease spread) [2,3]. Moreover, if a radical resection is feasible, without an (effective) adjuvant treatment, for these patients the risk of relapse can approach or exceed 90% within 5 years [4,5].

Starting from this evidence, highlighting the need for a safe and useful postoperative approach, several strategies have been investigated to improve the outcome in this setting.

## 2. Methods

This critical review evaluates evidence regarding adjuvant therapies that have received regulatory licensing from the Food and Drug Administration (FDA) or European Medicines Agency (EMA), or are currently recommended—or at least considered for selected clinical contexts—by the National Comprehensive Cancer Network (NCCN) and European Society of Clinical Oncology (ESMO) clinical practice guidelines. Even though adjuvant anti-PD-1 therapy is currently approved for fully resected stage II melanoma, this setting is outside the scope of this work. Consequently, stage II patients—as well as those with non-cutaneous forms of the disease—were excluded.

To ensure clinical relevance, comparative accuracy. and academic rigor, articles and documents were selected based on the following protocol.

Inclusion Criteria: 1. Peer-reviewed clinical trial publications (phases II and III), systematic reviews, and meta-analyses or other relevant work present in PubMed Database on adjuvant therapies for stage III and IV cutaneous melanoma, starting from those listed in current NCCN and ESMO guidelines at the time of submission of this work (4 May 2026). 2. Official regulatory approval regulatory documents, drug labels, and safety updates published by the FDA and EMA. 3. Studies published in the English language.

Exclusion Criteria: 1. Studies focusing on the neoadjuvant setting, localized surgical techniques, or therapies for non-cutaneous (e.g., uveal or mucosal) and stage II melanoma.

Instead of merely compiling a chronological narrative of drug approvals, the selected literature and clinical guidelines were analyzed using an adapted critical evaluation framework. For each therapeutic agent, the primary relevant clinical trials were described, and their integration into the corresponding guideline recommendations was evaluated. Furthermore, specific consideration was given to the application criteria and the unresolved questions regarding their implementation in real-world clinical practice.

## 3. Adjuvant Radiation Therapy (RT)

In resected stage III and IV melanoma, adjuvant RT concerns essentially the prevention of locoregional relapse (through tumor bed or nodal basin irradiation) and of intracranial recurrence after brain metastasectomy, respectively.

Primary tumor bed RT

The deployment of RT in this context is very limited, and—according to NCCN guidelines—could be considered after margin-negative surgical resection in the presence of certain clinical pathological predictors of local recurrence. These include:

- Primary T4 stage melanoma presenting in the head and neck area with neurotropism.
- Distinct desmoplastic histological subtypes.
- Pathologically narrow or close surgical margin.

Nodal basin RT

The use of radiation therapy (48 Gy in 20 fractions) vs. observation after a therapeutic lymphadenectomy was tested prospectively on 250 patients at risk of lymph-node field relapse (defined on the basis of nodal involvement) in a single randomized controlled trial (RCT). This strategy significantly reduced the probability of first local relapse (primary endpoint, HR 0.54; 95% CI 0.33–0.89; *p* 0.021), but did not impact relapse-free survival (RFS) (70 vs. 73 events, HR 0.91, 95% CI 0.65–1.26; *p* 0.56) or overall survival (OS) (59 vs. 47 deaths, HR 1.37, 95% CI 0.94–2.01; *p* 0.12) and was associated with a higher risk of adverse events (mostly radiation dermatitis, but also pain and nerve damage) [6]. Due to these data not generating consensus among panelists, the use of radiation therapy in this context in not recommended by NCCN and ESMO guidelines currently, even if this option could be discussed by the multidisciplinary team as part of the adjuvant strategy in selected patients. Among risk factors to take into account, the NCCN lists:

- Gross and/or microscopic extracapsular extension of the tumor.
- Involvement of at least one parotid node.
- Involvement of two or more cervical or axillary lymph nodes.
- Involvement of three or more inguinofemoral lymph nodes.
- A single pathologically involved node measuring ≥ 3 cm in the cervical or axillary regions or ≥4 cm in the inguinofemoral region.

Post-brain metastasectomy RT

Although the use of radiotherapy (RT) after surgery on brain metastases remains in common use, evidence is limited and largely based on prospective randomized controlled trials (RCTs) including several primary malignancies and comparing observation to adjuvant whole-brain radiation therapy (WBRT), stereotactic radiosurgery (SRS), or fractionated stereotactic RT (SRT). In this body of research, melanoma patients were not well represented (in total, fewer than 60 and 18 in the largest of these trials), and the histology-specific outcomes were not separately reported. Here, WBRT or SRS/SRT seemed to reduce the risk of intracranial progression with no statistically relevant effect on OS or preservation of functional independence. Concerning the use of SRS/SRT in this setting, even if it is appealing for the possibility of reducing the risk of neurocognitive toxicities associated with WBRT, there are no useful data from prospective randomized trials. For this lack of evidence, brain adjuvant RT should be considered for these patients on a case-by-case basis, taking into account the prolongation of their survival given by the introduction of modern medical therapies and their consequent higher risk of long-term neurocognitive toxicity, given above all by WBRT, which is not recommended [7,8].

Systemic therapies show more promise than local approaches in the adjuvant setting for this histology, probably because of the consistent ability of melanoma cells to spread both by the lymphatics and by the bloodstream, with the consequent tendency to metastasize beyond the regional basin and the resulting need for broad protection against cancer dissemination [9].

## 4. Medical Therapy

When this review was written, standard-of-care systemic adjuvant therapy for stage III and IV melanoma according to the US Food and Drug Administration (FDA) and EMA approvals and taking into account NCCN and ESMO guidelines could be summarized as follows and is presented in Table 1.

➢ Stage III: nivolumab or pembrolizumab (regardless of BRAF mutation).
➢ Stage III: dabrafenib + trametinib (only if BRAF V600 mutation present).
➢ Stage III: consider ipilimumab after total lymphadenectomy, limited to cases of nodal recurrence after previous exposure to an anti-PD-1 (regardless of BRAF mutation) (only by FDA, not EMA).
➢ Stage IV: nivolumab (regardless of BRAF mutation) [8].

**Figure 1:**
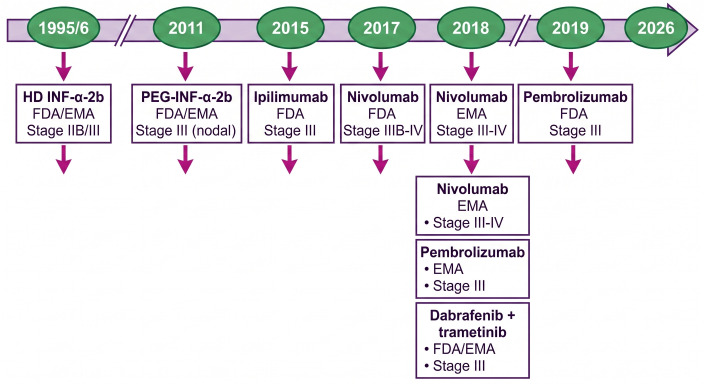
Timeline of FDA and EMA approvals of adjuvant systemic therapy for resected stage III and IV melanoma.

We will now again go through the main studies that led to these regulatory licensing, also covering the evidence regarding interferon (IFN), the first and only adjuvant therapy approved by the FDA and EMA until 2015. A timeline of FDA and EMA approvals in the melanoma adjuvant setting is presented in Figure 1.

### 4.1. Interferon

Even though it has been investigated in several trials in the adjuvant setting also, our interest applies above all to those including testing of interferon on target stages.

The first data suggesting the possibility of using this agent in the context of an earlier stage with respect to metastatic unresectable disease came from the ECOG 1684 study (published in 1996): in stage IIB–III patients, high-dose IFN-α-2 b (HD-IFN) significantly improved RFS from 0.98 to 1.72 years (*p* 0.0023, one-sided) and OS from 2.78 to 3.82 years (*p* 0.0237, one-sided) compared to observation at a median follow-up of nearly 7 years in surviving subjects. It is clear from observing reported data that RFS impact was more consistent than the benefit on OS, which even seemed to be lost in subsequent updates of the study at a doubled follow-up interval and in other clinical studies evaluating this therapy in this setting. The treatment regimen in the experimental arm was based on the administration of IFN-α-2 b at a dose of 20 MU/m^2^ for 5–7 days a week for 4 weeks followed by a subcutaneous administration of 10 MU/m^2^ three times for week for 48 weeks. Unfortunately, this positive survival impact pertained to a restricted group of subjects, and the treatment could not be defined manageable considering safety profile [10,11].

Despite that, considering that HDI was the very first regimen to show signals of activity against this cancer in the adjuvant setting also, it was approved for radically resected high-risk stage IIB–III melanoma by the FDA in 1996 and by the EMA in 2004.

Nevertheless, almost 20 clinical trials continued to assess different IFN schedules in resected high-risk patients, with the aim of investigating the need for dealing with HD-IFN side effects and preserving or increasing treatment benefits.

Several studies assessed the use of low-dose interferon (LD-IFN) adjuvant therapy in melanoma compared to observation, with conflicting results.

Among these, the Scottish Melanoma Group study was likely the first to also include stage III disease. It was initially presented as a personal communication by M.C. Cornbleet in 1995 and subsequently published in 2000. This trial, whose aim was to evaluate IFN-α-2 b at a dose of 3 MU thrice weekly for six months, failed to demonstrate a significant difference between the two arms regarding RFS or OS. The authors noted that while a clinical improvement was foreseen, the lack of statistical significance could be explained by the underpowering of the study. This resulted from scarce enrollment, as the trial included only 96 patients and closed prior to reaching its accrual target [12].

After this work, the randomized controlled WHO Melanoma Program Trial 16 and AIM-HIGH trial evaluated the deployment of INF-α-2 b 3 in radically resected stage IIB and stage III at a dose of MUI 3 days per week for 2 years and 3 years, respectively, showing no significant difference in OS or RFS between the interferon-treated and control arms [13,14]. Conversely, Garbe et al. reported a significant positive impact on both OS and RFS following two years of adjuvant IFN-α-2 b (3 MIU thrice weekly) in melanoma patients with regional lymph-node metastasis. When compared to surgery alone, four-year OS was 59% versus 42% (*p* 0.0045) and four-year RFS was 39% versus 27% (*p* 0.018). As such, the OS benefit was not only statistically significant but also larger than that of risk of relapse. Moreover, the addition of dacarbazine chemotherapy (850 mg/m^2^ every 4–8 weeks for two years) not only failed to provide additional benefit over low-dose IFN (LD-IFN) but appeared to negate the positive effects of the interferon therapy itself [15].

The authors of this last study suggested that these controversial results may be interpreted in part as the effect of chance in small studies, which can lead to the underestimation or overestimation of treatment effects. However, we believe that the evidence of this benefit may be at least partially linked to the exclusive inclusion of patients with stage III disease. These patients have a more unfavorable prognosis, which increases the likelihood of identifying a positive trend in an effective treatment, thereby confirming the crucial role of patient selection in postsurgical strategies.

Intermediate-dose IFN (IDI) was investigated in two RCTs. In EORTC-18952, the 13-month IFN group showed a 3.2% increase in rate of DMFI at 4.5 years (HR 0.93 [0.75–1.16] *p* 0.48) and no extension of overall survival (HR 0.97 [0.77–1.21]; *p* 0.73) with respect to observation. The 25-month IFN group showed a 7.2% increase in rate of DMFI (HR 0.83, 97.5% CI 0.66–1.03, *p* 0.05) and a 5.4% improvement in overall survival (HR 0.85, 97.5% CI 0.68–1.07, *p* 0.12). These findings suggested no effects on DMFI or overall survival of IDI in stage IIB/III melanoma [16]. The same inconsistent RFS and OS benefit of IDI was confirmed in the Nordic IFN trial, where stage IIB–IIC and III melanoma patients were randomly assigned to: a. observation; b. 4 weeks of induction followed by 12 months of maintenance therapy; or 4 weeks of induction and 24 months of maintenance [17]. Trials using intermediate-dose regimens have not been conducted very often, as the focus has mainly been on high- and low-dose regimens.

In 2000, the ECOG 1690 study confirmed in stage IIB–III the superiority of standard HD-IFN compared to standard low-dose IFN-α-2 b (LD IFN) for two years in terms of RFS with respect to observation, but not when analyzing OS [18].

Moreover, based on the early separation of RFS and OS curves in the E1684 trial (which could be justified by the effect of the induction phase) and on the fact that some toxicities, in particular fatigue and depression, which heavily impact quality of life, were cumulative during the year of therapy [10], it was tested whether within the HD-IFN regimen, the addition of 48 weeks of subcutaneous injections of adjuvant interferon-α-2 b maintenance therapy could provide further benefits over intravenous induction therapy alone for four weeks.

Results presented by the Hellenic Cooperative Oncology Group (HeCOG) in 2009 showed non-inferiority in terms of OS and RFS in resected stage IIB, IIC, and III melanoma for the induction-only therapy compared to the complete HD-IFN scheme, suggesting that maintenance therapy did not provide additional benefit on the assessed outcomes [19]. These results were apparently in contrast with those reported by Agarwala et al. in 2011 [20] and by Payne et al. in 2014 [21]. Indeed, both works assessed the use of a IV induction phase of a standard high-dose IFN regimen, comparing it to observation or to the complete standard high-dose IFN regimen: no RFS and OS benefit were highlighted with respect to observation alone, and a negative impact of these outcomes was recorded when the induction was set against the whole plan of treatment [20,21]. However, it was postulated that these disparities in terms of results could be linked to the different therapeutic schemes evaluated: in particular, in the first cited trial, Pectacides et al. randomly assigned participants to receive IFN-α-2 b at a dose of 15 MU/m^2^ for 5–7 days a week for 4 weeks alone or followed by subcutaneous administration of 10 MU (flat dose) three times for week for 48 weeks, and this could translate to reduced drug exposure (not quantifiable in the maintenance phase), which makes interpretation of the data complex and less immediate than for the other two works, in addition to potentially underestimating the impact of the maintenance phase.

Moreover, an Italian Melanoma Intergroup (IMI) phase III RCT and a trial of the Dermatologic Cooperative Oncology Group suggested the possibility of using shorter, but more intensive HDI regimens (INF-α-2 b 20 MU/m^2^/day for 5 days per week 4 weeks given every other month for four courses or every fourth month for three courses) in stage III resected melanoma patients, which resulted in more feasible and not more toxic than conventional HDI without differences in efficacy (RFS and OS) [22,23].

To address the issue of the doubt concerning the survival impact of interferon in the adjuvant setting, systematic reviews and meta-analyses were performed.

Even if not clarifying the optimal schedule of treatment, an 18-RCT meta-analysis published by Cochrane in 2013 showed an improvement in disease-free survival (HR 0.83; 95% CI 0.78–0.87; *p* < 0.00001) and a significant, but quantitatively limited benefit in overall survival (HR 0.91; 95% CI 0.85–0.97; *p* 0.003) that favored the use of adjuvant IFN in stage II–III melanoma patients [24].

A meta-analysis of all randomized trials concerning the deployment or not of IFN-α-2 b in high-risk melanoma in the adjuvant setting (15 trials and 7744 patients) was then performed in 2017 by the International Melanoma Meta-Analysis Collaborative Group (IMMCG), demonstrating a significant benefit for both event-free survival (EFS) (HR 0.86, 95% CI 0.81–0.91; *p* < 0.00001) and OS (HR 0.90, 95% CI 0.85–0.97; *p* 0.003), with an absolute gain of 3.5–2.7% in EFS and 3.0–2.8% in OS. Notably, among the clinical and pathological elements tested (including age, gender, stage, and schedule of administration), the only association with an impact on outcomes in cases of treatment was ulceration, suggesting consideration of this adjuvant treatment first for subjects with ulcerated primary tumors [25].

From the evidence presented, while a benefit on survival outcomes seemed to be confirmed (even if limited), no single clinical study and none of the meta-analyses conducted help to identify the ideal dose, the most advantageous schedule, or the optimal duration of IFN-α-2 b treatment in the adjuvant therapy of intermediate- to high-risk melanomas. However, a positive trend for the risk of relapse, and to a lesser extent the risk of death, was almost always present, with an estimated absolute difference in survival of about 3%, a relative benefit ranging from 10 to 17%. These considerations justified its use as the only adjuvant treatment approved by the FDA until the introduction of immunocheckpoint inhibitors.

### 4.2. Ipilimumab

Ipilimumab is a fully human monoclonal antibody that blocks cytotoxic T-lymphocyte antigen 4 (CTLA-4). It was the first immunocheckpoint inhibitor approved for a solid tumor and the first agent to demonstrate improved overall survival in patients with previously treated metastatic melanoma [26]. Following this success in unresectable advanced disease, it was subsequently evaluated in the adjuvant setting.

The landmark EORTC 18071 trial enrolled 951 patients with resected stage III melanoma (AJCC 7th ed.), comparing high-dose ipilimumab (10 mg/kg) with placebo. Enrollment was limited to nodal basin involvement. Consequently, subjects with in-transit metastases or a nodal tumor burden less than 1 mm (specifically for stage IIIA and N1 a disease) were excluded. Complete lymphadenectomy was required following radical excision of the primary tumor. The treatment schedule in the intervention arm consisted of intravenous ipilimumab at a dose of 10 mg/kg every 3 weeks for four courses, followed by maintenance doses every 3 months for up to 3 years (ipi10). The primary endpoint was RFS [27].

After a median follow-up of 6.9 years, the ipi10 regimen confirmed significant superiority across both primary and secondary endpoints. Specifically, long-term follow-up revealed a 7-year RFS rate of 39.2% for ipilimumab versus 30.9% for placebo (HR 0.75; *p* < 0.001). Similar gains were observed in 7-year OS (60.0% vs. 51.3%; HR 0.73; *p* 0.002) and DMFS (44.5% vs. 36.9%; HR 0.76; *p* 0.002) [28]. Despite the significant interest generated by the efficacy data of this drug, safety concerns played a pivotal role in limiting its clinical adoption in the adjuvant setting. Grade 3 or 4 adverse events (AEs) occurred in 54.1% of patients in the treatment group (including 41.6% immune-related AEs [irAEs]) compared to 26.2% in the control arm (where irAEs accounted for 2.7% of cases). This translated into a safety-related discontinuation rate of 52% in the ipilimumab arm. Consequently, only 29% of patients completed one year of maintenance therapy [28]. For these reasons, and given the heightened importance of patient safety in the adjuvant context, the FDA approved ipilimumab for resected stage III melanoma, whereas it remains unapproved for this indication in Europe.

Given that ipilimumab was already approved at a dose of 3 mg/kg (ipi3) for unresectable metastatic melanoma, the 2015 adjuvant approval of the 10 mg/kg dose (ipi10) created a clinical necessity to compare the safety and efficacy of these two dosages against the existing standard of care, high-dose interferon (HDI). To address this issue, the Intergroup E1609 trial randomized 1670 patients with radically resected stage IIIB, IIIC, M1 a, or M1 b melanoma (AJCC 7th ed.) into three arms: (a) ipi3, (b) ipi10 (both administered intravenously every 3 weeks for four induction doses, followed by maintenance every 12 weeks for up to 60 weeks), or (c) standard intravenous HDI. OS and RFS served as co-primary endpoints. The comparative efficacy analysis revealed that only the lower dose of ipilimumab (ipi3) provided a statistically significant improvement in 5-year OS when compared against HDI (72% vs. 67%; HR 0.78; *p* 0.044). Moreover, this regimen showed a strong trend that approached significance in terms of median RFS (4.5 vs. 2.5 years; HR 0.85; *p* 0.065). Conversely, the comparison between ipi10 and HDI yielded only exploratory trends in favor of ipilimumab for both 5-year OS (70% vs. 65%; HR 0.88) and median RFS (3.9 vs. 2.4 years; HR 0.84) without achieving statistical significance. Exploratory comparisons between the two ipilimumab dose levels showed no significant differences in OS or RFS [29].

Observing safety, overall grade ≥ 3 AEs were nearly twice as common with HDI (78.8%) compared to ipi3 (38.6%), while ipi10 demonstrated an intermediate risk of toxicity (57.9%). Between the two ipilimumab arms, the frequency of overall ≥ G3 toxic events significantly differs (*p* 0.001) from immune-related ≥ G3 AEs (28.5% ipi3 vs. 46.3% ipi10; *p* < 0.001). Moreover eight toxicity deaths occurred in the ipi10 arm compared to only two to three in the other two treatment groups. Discontinuation rates were 88.5%, 64.9%, and 61.6% for ipi10, ip3, and HDI respectively [29].

The E1609 trial’s efficacy findings must be interpreted within the context of its active control arm and post-progression therapy patterns. Notably, the significant OS improvement seen with ipi3 compared to HDI, despite a non-significant RFS trend, could be interpreted as: (a) the result of a limited sample size, which can make the detection of differences more difficult; (b) the result of a more lasting effect of ipi3, with a consequently greater OS benefit; and (c) the effect of the high rate of crossover to modern immunotherapies in the HDI cohort (86.2%). Conversely, the lack of significant benefit in the ipi10 arm was likely influenced by its prohibitive toxicity profile, which not only reduced treatment exposure but also limited the feasibility of subsequent salvage therapies upon recurrence. The authors concluded that ipi3 offered a distinct survival advantage over HDI, with efficacy gains that sufficiently justified its increased toxicity. In contrast, while ipi10 provided a comparable therapeutic benefit compared to ipi3, it was associated with a disproportionate and unacceptable escalation in toxic effects [29]. Although these data suggested a superior cost-effectiveness ratio for ipi3 relative to both ipi10 and HDI, the simultaneous approval of anti-PD-1 and BRAF-targeted therapies—which offer superior efficacy and safety profiles—ultimately precluded the regulatory adoption of this regimen. Consequently, current NCCN guidelines recommend ipilimumab in the adjuvant setting only for cases of nodal recurrence following previous anti-PD-1 exposure and at the FDA-approved dosage of 10 mg/kg every 3 weeks for four doses, followed by 10 mg/kg every 12 weeks for up to 3 years (EORTC 18071 regimen).

### 4.3. Anti-PD-1 Monotherapy

Programmed cell death protein 1 (PD-1) is an immunocheckpoint that regulates effector T-cell activation through a mechanism distinct from CTLA-4. It functions via engagement with its ligand (PD-L1), which is expressed in both peripheral and cancerous tissues. Anti-PD-1 antibodies interfere with this binding, resulting in enhanced T-cell activation, improved efficacy, and a more durable clinical benefit compared with ipilimumab in unresectable melanoma [30]. Mirroring success in the advanced setting, two anti-PD-1 agents—nivolumab and pembrolizumab—have been approved by the FDA for the adjuvant treatment of resected stage III melanoma.

#### 4.3.1. Nivolumab

The CheckMate 238 trial served as a landmark head-to-head comparison between PD-1 and CTLA-4 inhibition in the adjuvant setting. Researchers enrolled 906 patients with resected high-risk melanoma (stages IIIB–IV per AJCC 7th edition), excluding those with stage IIIA disease. Participants were randomized to receive a one-year course of either nivolumab (3 mg/kg every 2 weeks) or a high-dose ipilimumab regimen (10 mg/kg every 3 weeks for four doses, then every 12 weeks). Notably, the study population included patients with resected M1 disease and those with in-transit metastases, provided they had undergone mandatory complete surgical resection [31].

Concerning the primary endpoint of the trial, recurrence-free survival (RFS), at a minimum follow-up of 18 months, the study highlighted the superiority of nivolumab over ipilimumab. The 12-month RFS rate was 70% for the anti-PD-1 arm compared to 60.8% for the anti-CTLA4 arm (HR 0.65; *p* < 0.001). Nivolumab efficacy was consistently observed across various clinicopathological factors and was independent of BRAF mutational status, disease stage, or the nature of nodal involvement. RFS results for the ipilimumab arm were consistent with those of the EORTC trial, in which ipilimumab was administered for longer (3 years vs. 1 year) including stage IIIA patients, but excluding those with stage IV disease. Additionally, an exploratory evaluation suggested a benefit for nivolumab regarding distant metastasis-free survival (DMFS) [31].

Nivolumab also demonstrated a better safety profile. Grade 3–4 treatment-related adverse events occurred at nearly one-third the rate of the ipilimumab group (approximately 15% vs. 46%), leading to a significantly lower probability of discontinuation due to safety concerns (7.7% vs. 41.7%). Furthermore, quality-of-life scores appeared more favorable in the anti-PD-1 arm [31].

These results were reinforced by a 4-year median follow-up published in 2020. Nivolumab showed a sustained benefit with a 4-year RFS rate of 51.7% vs. 41.2% for ipilimumab (HR 0.71; *p* 0.0003). This benefit was maintained in most subgroups, with the exception of mucosal primary and resected stage M1 c patients. A post hoc analysis of 155 patients with in-transit metastases without nodal involvement found an RFS advantage similar to that of the intention-to-treat population, with a preserved benefit of anti-PD-1 over ipilimumab also. While indirect comparisons across trials are methodologically limited, these results suggest that nivolumab compares favorably to other adjuvant options, such as pembrolizumab or dabrafenib plus trametinib [32].

Regarding DMFS in stage IIIB–C disease, the 4-year data remained more favorable for nivolumab (59.2% vs. 53.3%; HR 0.79), with the median DMFS not yet reached in the nivolumab arm. The primary report of overall survival (OS) was also released; however, it was performed with less than 75% of the planned power, as only 211 of the 302 required events had occurred. Consequently, 4-year survival was similar between groups: 77.9% for nivolumab and 76.6% for ipilimumab (HR 0.81; *p* 0.31). This lack of statistical significance in OS likely reflects the impact of effective subsequent therapies—primarily anti-PD-1 agents in the ipilimumab arm—which may confound OS results and lead to high survival rates in both groups [32].

The 2025 update (minimum follow-up of nearly 9 years) confirmed a 9-year RFS of 44% vs. 37% in the nivolumab vs. ipilimumab arm with a median of 61.1 vs. 24.2 months (HR 0.76; 95% CI 0.63–0.90) and a DMFS of 54% vs. 48%, with a median of 9 years vs. 83.8 months, respectively (HR 0.81; 95% CI 0.65–1), though OS still did not significantly differ between treatments (HR 0.88; 95% CI 0.69–1.11). Moreover, nivolumab was also confirmed favored in terms of safety, with G3 or G4 AEs threefold less frequent (14.4%) than in ipilimumab cohort (45.9%) and a significantly lower toxicity discontinuation rate (9.7% vs. 42.6%) [33].

The remarkably consistent 9-year data from CheckMate 238 solidify nivolumab’s role as a cornerstone of adjuvant therapy. By providing sustained RFS and DMFS benefits with a toxicity profile far more manageable than high-dose CTLA-4 inhibition, it remains a standard of care for high-risk resected melanoma.

#### 4.3.2. Pembrolizumab

The efficacy of adjuvant pembrolizumab for resected melanoma was established in the landmark EORTC 1325/KEYNOTE-054 trial. A total of 1019 patients with radically resected stage IIIA, IIIB, or IIIC melanoma (AJCC 7th ed.) were randomized to receive either 200 mg of pembrolizumab or a placebo every three weeks for up to one year (maximum 18 infusions). Notably, the trial excluded stage IIIA patients with a sentinel node tumor burden smaller than 1 mm in diameter. Furthermore, complete lymphadenectomy was required for nodal disease, while patients with in-transit metastases without nodal involvement were excluded [34].

Initial results after a median follow-up of 15 months showed that pembrolizumab significantly improved recurrence-free survival (RFS) compared to placebo (1-year RFS: 75.4% vs. 61.0%; HR 0.57; 98.4% CI 0.43–0.74; *p* < 0.001). This represented a 43% reduction in the risk of relapse, an advantage that was consistent across clinicopathological subgroups. Moreover, distant metastases developed in 15.2% of the pembrolizumab group compared to 27.3% in the placebo cohort [34].

Treatment-related grade 3–5 adverse events (AEs) were reported in 14.7% of patients receiving pembrolizumab versus 3.4% for placebo. One treatment-related death due to myositis occurred in the experimental arm. Immune-related AEs occurred in 37.3% of the pembrolizumab group compared to 9.0% for placebo. Consequently, adjuvant pembrolizumab appears significantly less toxic than high-dose ipilimumab (45.9% grade 3–4 AEs) and comparable to nivolumab (14.4%) [34].

This therapeutic benefit proved durable: at the 3.5-year update, pembrolizumab maintained superior RFS (59.8% vs. 41.4%; HR 0.59; *p* < 0.0001) and distant metastasis-free survival (DMFS) (65.3% vs. 49.4%; HR 0.60; *p* < 0.0001). These results were consistent across all subgroups, including PD-L1 status, *BRAF* mutation, and disease stage. The observed HRs in this trial (0.59–0.60) appeared more favorable than those reported in CheckMate 238 (0.71–0.79), likely due to the use of a placebo control rather than an active ipilimumab comparator. Additionally, results in the *BRAF*-mutated subgroup, similar to or slightly better than those reported for adjuvant dabrafenib plus trametinib, confirmed the potential of anti-PD-1 therapy in this population [35].

Long-term data from the 7-year analysis confirmed the sustained impact of PD-1 inhibition, with a 7-year RFS rate of 50% versus 36% for placebo (HR 0.63; 95% CI 0.53–0.74). This survival advantage remained consistent across all strata, including *BRAF* and PD-L1 status. Overall survival (OS) data remain immature, partly due to the crossover design and the availability of effective salvage treatments upon recurrence [36].

The efficacy of pembrolizumab was further validated against previous standards of care (SOC) in the SWOG S1404 clinical trial, which enrolled 1301 patients with resected stage IIIA–IV melanoma between December 2015 and October 2017. Participants were randomized to receive either pembrolizumab or the investigators’ choice of SOC. In the initial phase of the trial, SOC consisted of high-dose interferon (HD-IFN). Following the FDA approval of ipilimumab, the control arm was expanded to include either ipilimumab (10 mg/kg) or HD-IFN [37].

At a median follow-up of 47.5 months, pembrolizumab was associated with significantly superior recurrence-free survival (RFS) compared to the SOC (HR 0.77; 99.62% CI 0.59–0.99; *p* 0.002). Furthermore, pembrolizumab demonstrated a more favorable safety profile: the rate of grade 3–5 adverse events (AEs) was only 19.5%, a marked reduction compared to the 71.2% observed with interferon alpha-2 b and 49.2% with ipilimumab. Despite the RFS benefit, no statistically significant improvement in overall survival (OS) was observed in any of the patient population (HR 0.82; 96.3% CI 0.61–1.09; *p* 0.15) [37].

As demonstrated by these pivotal trials, both anti-PD-1 agents (nivolumab and pembrolizumab) have proven more effective than previous standards in terms of RFS while offering a more manageable toxicity profile, though OS data remain immature or inconclusive. To date, no head-to-head trials have directly compared these two anti-PD-1 molecules in the adjuvant setting. However, an analysis of data from CheckMate 238 and S1404—which are broadly comparable due to the inclusion of stage IV disease and the use of active control arms—reveals no striking differences in performance. This consistency is expected, given the shared mechanism of action of these molecules and the evidence already established in the metastatic setting.

### 4.4. Nivolumab Combined with Ipilimumab

Currently, the combination of nivolumab (1 mg/kg) and ipilimumab (3 mg/kg) every three weeks for four doses, followed by nivolumab maintenance (nivo1–ipi3), represents a benchmark in the treatment of metastatic melanoma. This regimen is associated with a median overall survival (OS) of 72.1 months compared with 36.9 and 19.9 months for nivolumab and ipilimumab monotherapies, respectively. Although the pivotal trial was not powered for a direct comparison between the combination and nivolumab single-agent therapy, the clinical benefit of the doublet is clear [38].

The CheckMate-915 trial assessed whether the addition of ipilimumab to nivolumab could improve relapse-free survival (RFS) in the adjuvant setting. The study randomized 1883 patients to receive either a low-dose ipilimumab regimen (1 mg/kg every 6 weeks) plus nivolumab (240 mg every 2 weeks) or nivolumab monotherapy (480 mg every 4 weeks) for up to one year [39]. Despite selecting this low-dose schedule to enhance efficacy while minimizing the toxicity of the doublet, no significant difference in 2-year RFS was observed between the two groups at a median follow-up of 23.7 months (64.6% for the combination vs. 63.2% for monotherapy; HR 0.92; 95% CI 0.77–1.09; *p* 0.269). Conversely, grade 3–4 adverse events (AEs) and discontinuation rates more than doubled in the combination group (32.6% vs. 12.8% and 34.6% vs. 11.3%, respectively) [39]. It was initially suggested that the lack of benefit in the combination arm might be due to a shorter median duration of therapy (7.6 vs. 11.1 months) and a lower cumulative nivolumab exposure. However, subsequent analyses showed these factors were insufficient to explain the results, given the similarity in 24-month RFS rates between patients who discontinued early due to toxicity and those who completed the full course. Instead, the failure to improve RFS appears to be a consequence of the low and infrequent ipilimumab dosing, which prevented adequate drug exposure [40]. Multiple studies have confirmed that both ipilimumab activity and toxicity are dose-dependent in both metastatic and adjuvant settings [40,41,42]. In this context, the CheckMate-511 trial demonstrated that a “flipped dose” schedule (nivo3/ipi1) resulted in a lower frequency of grade 3–4 AEs during the first four cycles compared with the standard nivo1–ipi3 regimen, while suggesting similar 3-year OS and PFS rates, although the trial’s non-inferiority design focused primarily on safety [43,44].

The German phase II IMMUNED study evaluated adjuvant combination therapy in metastatic melanoma patients with no evidence of disease (NED) after surgery or radiotherapy. Patients were randomized to nivo1–ipi3 (followed by nivolumab maintenance), nivolumab monotherapy, or placebo for up to one year. The primary endpoint was RFS, and crossover to nivolumab was allowed in cases of recurrence during double placebo treatment. Even if exploratory analyses were done, the study design was not powered to compare the two active arms of the trial, as it started before nivolumab approval as adjuvant therapy (2015) [45]. At the 2022 analysis, with a median follow-up of 49.2 months, the combination demonstrated a significant 4-year RFS advantage over placebo (64.2% vs. 15.0%; HR 0.25; *p* <0.0001) and a 4-year OS benefit (83.8% vs. 63.1%; HR 0.41; *p* 0.040). While nivolumab monotherapy improved 4-year RFS compared to placebo, it did not achieve a significant OS benefit (72.6% vs. 63.1%; HR 0.75; *p* 0.44). As in previous trials, grade 3–4 AEs were significantly more frequent with the combination (71% vs. 29%). Based on these results, the combination of nivolumab and ipilimumab should be considered for approval in stage IV melanoma with no evidence of disease. On the basis of the evidence of efficacy of this trial, although probably also the post-recurrence use of anti-PD-1 based therapy in patients in the placebo arm could affect the limited evidence of impact of nivolumab monotherapy in terms of OS with respect to placebo, the RFS and OS benefit of combination should support its approval in stage IV melanoma with no evidence of disease [46].

### 4.5. BRAF-Targeted Therapy

The efficacy and safety of BRAF-targeted therapy in the adjuvant melanoma setting were assessed in two prospective, double-blind, randomized controlled trials.

The COMBI-AD trial explored the impact of a 12-month course of either dual kinase inhibition or placebo in 870 patients with completely resected (including radical lymphadenectomy) stage III (AJCC 7th ed.) BRAF V600 E- or V600 K-mutated melanoma. Patients with stage IIIA disease and minimal nodal involvement (metastasis of ≤1 mm) were excluded. The treatment arm received oral dabrafenib (150 mg twice daily) plus trametinib (2 mg once daily). The primary endpoint was relapse-free survival (RFS), with overall survival (OS), distant metastasis-free survival (DMFS), and safety as secondary endpoints [47].

After a median follow-up of 2.8 years, the study demonstrated that combined targeted therapy significantly reduced the risk of recurrence, with 3-year RFS rates of 58% vs. 39% for placebo (HR 0.47; 95% CI 0.39–0.58; *p* <0.001). Combination therapy also reduced distant metastasis rates (25% vs. 35%; HR 0.51; 95% CI 0.40–0.65; *p* <0.001). Regarding OS, 3-year rates were 86% and 77% in the combination and placebo groups, respectively (HR 0.57; 95% CI 0.42–0.79; *p* 0.0006). Although clinically relevant, this did not meet the stringent prespecified interim analysis threshold of *p* 0.000019 [47].

Safety signals were consistent with those reported in the metastatic setting. At least one adverse event (AE) was reported by 97% of patients in the combination group compared with 88% in the placebo group. The most frequent AEs in the combination arm were pyrexia (any grade 63%; G3–4 5%), fatigue (any grade 47%; G3–4 4%), and nausea (any grade 40%; G3–4 <1%). Grade 3–4 treatment-related AEs, events leading to permanent discontinuation, and dose reductions were present in 41%, 26%, and 38% of the combination group, respectively, versus 14%, 3%, and 3% in the placebo group. Serious adverse events occurred in 36% of patients in the combination-therapy group and 10% of cases in the placebo group. Taking into account the oncological diagnoses, a new primary melanoma, cutaneous squamous-cell carcinoma (SCC) or keratoacanthoma (KA), basal-cell carcinoma, and non-cutaneous cancers were reported in 3% (vs. 2%), 2% (vs. 2%), 4% (vs. 3%), and 2% (vs. 1%) of patients in the combination-therapy group (with respect to the placebo group) [47].

Long-term data at a median follow-up of approximately 8 years showed an 8-year RFS rate of 50% with dabrafenib plus trametinib versus 35% with placebo (HR 0.52; 95% CI 0.43–0.63). The 8-year DMFS rate was 64% in the combination arm compared with 53% in the placebo cohort (HR 0.56; 95% CI 0.44–0.71). On the other hand, no statistically significant impact on OS was confirmed, though a positive trend was suggested. Regarding relapse-free survival, dabrafenib plus trametinib offered comparable benefits in both BRAF V600 E and V600 K melanoma. However, overall survival trends for the V600 K subgroup were completely inverted compared to the V600 E cohort. This divergence suggests that adjuvant combination therapy might actually be disadvantageous for BRAF V600 K patients. Nevertheless, these results require careful interpretation due to the small V600 K sample and the potential confounding impact of highly effective immunocheckpoint inhibitor (ICI) salvage therapies. The latter could be explained by distinct cellular pathway aberrations and high mutational burdens in V600 K tumors, which may bypass BRAF inhibition, but render them more responsive to immunotherapy [48].

The phase III BRIM8 trial used hierarchical testing to compare disease-free survival in nearly 500 adults with resected BRAF V600-mutated melanoma. Patients were divided into cohort 1 (stage IIC–IIIB) or cohort 2 (stage IIIC) and randomized to vemurafenib (960 mg twice daily) or placebo for one year. The trial design required statistical significance in cohort 2 (*p* ≤ 0.05) to validate findings in cohort 1. At 30 months, cohort 1 results were deemed statistically non-significant due to a lack of significance in cohort 2. Grade 3–4 AEs occurred in 57% of the vemurafenib arm compared with 15% for placebo, primarily affecting the skin, liver, and joints [49].

Due to the primary endpoint failure and the increase in grade 3–4 AEs—particularly hyperproliferative cutaneous events—vemurafenib monotherapy is not FDA-approved for adjuvant use. Conversely, the superior efficacy and safety profile of the dabrafenib/trametinib doublet led to its FDA approval for the adjuvant treatment of stage III melanoma. Pivotal trials on adjuvant immunocheckpoint inhibitors and BRAF-targeted therapy in completely resected stage III–IV cutaneous melanoma are summarized in Table 2.

## 5. Some Open Questions Regardingn Adjuvant Treatment of Stage III–IV Cutaneous Melanoma

### 5.1. Impact of Substages and Evolution of Staging System

Pivotal trials on adjuvant ipilimumab (EORTC 18,071 and E1609), nivolumab (CheckMate 238), pembrolizumab (Keynote 054 and SWOG S1404), and dabrafenib + trametinib (COMBI-AD) completed their accruals before AJCC was revised. On the other side, in CheckMate 915 and MMUNED trials, assessing the use of ipilimumab + nivolumab in this setting, patients were classified according to the new staging system. Indeed, the 8th edition of AJCC was published in 2017 with the objective of better stratifying patients according to their risk of relapse or death [50]. Of note, one of the most important changes was the refinement of stage III subgrouping, as presented in Table 3. In particular, the new version included the stage IIID and provided a more accurate prognostication defining substage in the evaluation of tumor thickness, along with ulceration of primary and the variable (and also redefined) nodal involvement, introducing new pathological categories and a change in some pathological definitions [2]. As a consequence, patients could be assigned to a different subgroup when classified according to the new staging system, and even if no direct comparison were possible, a relevant improvement in stages IIIA, B and C melanoma survival when compared with the corresponding AJCC 7th edition stages emerged [51]. For these reasons, in order to assess the efficacy of the adjuvant treatments tested in the new stage III substages, updated analyses were performed.

Among registered trials, the first to present a post hoc analysis of RFS on the basis of baseline disease stage according to the AJCC 8th edition was COMBI-AD. Dabrafenib + trametinib improved RFS across all updated stage subgroups (HR 0.63 for stage IIIA, HR 0.48 for stage IIIB, HR of 0.50 for stage IIIC, and HR of 0.34 for stage IIID) compared with placebo at a median follow-up of 44 months and 42 months, respectively. However, even if a positive trend is suggested also for these subjects, that benefit seems not to have statistical relevance for the new stage IIIA (see below) [52].

A post hoc analysis of the CheckMate 238 trial evaluated the 4-year efficacy outcomes of adjuvant nivolumab versus ipilimumab by restaging patients. About 36.9% patients shifted from AJCC 7th edition stage IIIB to AJCC 8th edition stage IIIC, and only eight subjects (1.1% of the total study population) were reassessed as stage IIIA. Despite that, the superior efficacy of nivolumab over ipilimumab was fully preserved across both staging systems in terms of both RFS and DMFS across all evaluable subgroups.

The 4-year RFS rate for nivolumab-treated patients was 66.4% in AJCC 8th edition stage IIIB and 47.1% in stage IIIC (vs. 60% and 46.1% compared to ipilimumab). Similarly, the 4-year DMFS rates for nivolumab were 70.0% for stage IIIB and 55.7% for stage IIIC (vs. 67% and 52.5% compared to ipilimumab). While both treatment groups exhibited lower survival rates with increasing substage severity and respect to the previous classification, a stronger treatment effect was suggested in AJCC 8th edition stage IIIB patients (probably due to a large shift in higher-risk subjects to stage IIIC), the interaction tests confirmed no statistically significant differences between the hazard ratios of individual substages for RFS or DMFS, indicating a consistent treatment effect across the disease spectrum.

As established during the initial 1.25-year analysis, Keynote-054 trial data also confirmed that transitioning from AJCC-7 to AJCC-8 introduced significant substage migrations [53]. While the AJCC-8 system successfully carves out highly distinct prognostic categories at both ends of the risk spectrum, no predictive value regarding immunotherapy benefit was highlighted again. Long-term data at a 3-year median follow-up demonstrates a sustained, statistically RFS advantage for pembrolizumab over placebo across all substages. Specifically, 3-year RFS rates for pembrolizumab versus placebo were 82.6% vs. 67.4% for stage IIIA (HR 0.43); 70.4% vs. 51.7% for stage IIIB (HR 0.57); 59.6% vs. 35.2% for stage IIIC (HR 0.51); and 45.0% vs. 22.2% for the extremely high-risk stage IIID (HR 0.68). Notably, an outstanding benefit was observed in the best-prognosis subgroup (stage IIIA); however, interaction tests suggested no significant differences in hazard ratios across substages (*p* 0.90), and the wide confidence interval for stage IIIA (99% CI 0.13–1.43) underscores a lack of independent statistical significance within this specific cohort, even if could be primarily driven by the small sample and low number of events in this low-risk population rather than a true lack of treatment efficacy. Ultimately, these findings reinforce that utilizing pembrolizumab remains entirely valid irrespective of whether the AJCC-7 or AJCC-8 framework is applied [54].

In summary, these post hoc analyses across the COMBI-AD, CheckMate 238, and Keynote-054 trials demonstrate that while the AJCC 8th edition significantly refines prognostic stratification, it does not alter the therapeutic validity of modern adjuvant therapies. Across all three registered studies, formal interaction testing consistently confirmed that the relative treatment benefit of both targeted therapies and immunocheckpoint inhibitors is preserved across the newly defined disease spectrum. Even though the highly favorable stage IIIA cohorts occasionally exhibit wide confidence intervals or a lack of standalone statistical significance due to lower event rates, the overall clinical benefit remains clear. Ultimately, these data validate that the survival advantages of adjuvant anti-PD-1 agents and BRAF/MEK inhibitors remain robust and clinically meaningful, justifying their use irrespective of the AJCC staging edition applied.

### 5.2. Substage IIIA

The management of AJCC stage IIIA melanoma represents a significant clinical challenge due to the discordance between broad regulatory approvals for all stage III substages, despite the exclusion of all stage IIIA patients from CheckMate 238 (on nivolumab) [31] and of those with sentinel lymph node (SLN) metastases ≤ 1 mm in diameter in Keynote-054 and COMBI-AD (on pembrolizumab and dabrafenib + trametinib, respectively) [34,48]. Stage IIIA is characterized by a favorable prognosis relative to other stage III substages. Consequently, the absolute benefit of adjuvant therapy in this subgroup is potentially lower than in stage IIIB or IIIC, where the risk of relapse and death is substantially higher [55].

Efforts to validate efficacy in stage IIIA through subgroup analyses have been hindered. In both studies, however, substage IIIA was poorly represented, and very few events were observed, so the results are not statistically significant: for pembrolizumab. An HR of 0.93 was obtained with a confidence interval of 95% between 0.38 and 2.25; and for dabrafenib + trametinib, an HR of 0.83 with a 95% confidence interval of 0.36 to 1.91 [36,48].

On the basis of this evidence, actual clinical guidelines (NCCN, ESMO) suggest that adjuvant therapy for AJCC-8 stage IIIA should be considered carefully, using factors such as the 1 mm SLN burden threshold as a clinical benchmark to identify patients who are most likely to derive a meaningful absolute benefit from systemic treatment and always taking into account the risk of relevant toxicities associated with both the treatment regimens. Indeed, anti-PD-1 treatment is associated with serious or chronic AEs in about 15% and about 40% of patients [51], and dabrafenib + trametinib can cause severe pyrexia, retinal vein occlusion, and reduction in ejection fraction [47].

Among others, recent evidence from a large multicenter international study by Moncrieff et al. suggests that the current 1 mm threshold for adjuvant therapy in stage IIIA melanoma may be too restrictive. By identifying an optimal prognostic cut point of 0.3 mm for SN tumor deposits, the study demonstrates that patients with deposits face a significantly higher risk of recurrence and may derive more absolute benefit from systemic treatment than previously recognized. Conversely, the excellent prognosis of patients with deposits—comparable to SN-negative stage IB disease—supports a strategy of clinical observation for this specific subgroup, thereby sparing them from the risks of serious or chronic treatment-related toxicities [56]. Beyond the specific factors evaluated in this and other studies with similar objectives—namely to guide clinicians in their therapeutic decisions—it is clear that it is necessary to create integrated clinicopathological tools that include assessments of both metastasis and primary tumor (including the use of GEP panels) to reflect the complexity of the disease. These tools can contribute to better patient prognosis and consequently support oncologists in deciding whether or not to recommend adjuvant treatment in this subgroup of patients.

### 5.3. Safety Concerns

With their significant extension of RFS and DMFS, the introduction of immunocheckpoint inhibitors and BRAF/MEK targeted therapies has completely reshaped the landscape of adjuvant therapy for high-risk melanoma (stages IIB/C, III, and IV), becoming in this setting the standard of care. However, because adjuvant therapy is given to patients who are currently with no evidence of disease but at high risk of relapse, the conversation about toxicity is uniquely critical. Medical oncologists must heavily weigh the potential for cure, protecting patients from recurrence against the risk of causing severe or long-term, sometimes permanent, treatment-induced harm.

As is well known, the profile of toxicity in adjuvant melanoma therapy depends strictly on the mechanism of action of the chosen drug regimen.

As previously discussed, adjuvant immunotherapy primarily utilizes anti-PD-1 antibodies, such as pembrolizumab or nivolumab, that work by “unbraking” the patient’s own immune system to destroy microscopic remaining cancer cells, restoring T cell-mediated anti-tumor activity. These class-specific toxic events, sustained by a systemic breach in self-tolerance leading to autoimmune-like attacks on healthy tissues, are collectively called immune-related adverse events (irAEs).

Nivolumab displayed a significantly enhanced safety profile relative to high-dose ipilimumab. At the trial’s 18-month interim analysis, the risk of any-grade treatment-related adverse events (TRAEs) was 85% (vs. about 96% for ipilimumab), and grade 3 or 4 toxicity was restricted to 14.4% in the nivolumab group and 45.9% in the ipilimumab group. Furthermore, 42.6% of patients treated with ipilimumab discontinued therapy due to any adverse event (41.7% drug-related) compared to only 9.7% overall (7.7% drug-related) in the nivolumab arm.

The most frequent toxicities reported in at least 10% of patients in either arm revealed notable variations. Common any-grade toxicities in the nivolumab group included fatigue (34.5%) diarrhea (24.3%), pruritus (23.2%), and rash (19.9%). Grade 3–4 manifestations of these events remained low under nivolumab, such as diarrhea (1.5%), rash (1.1%), and elevations in alanine aminotransferase (1.1%). In contrast, ipilimumab induced higher rates of any-grade diarrhea (45.9%), rash (29.4%), hypophysitis (10.6%), and severe grade 3–4 elevated ALT and AST (5.7%). Treatment-related adverse events leading to discontinuation occurred in 7.7% of patients treated with nivolumab and in 41.7% of those in the ipilimumab arm [31].

In Keynote-054, TRAEs of any grade occurred in 77.8% of patients in the pembrolizumab arm (vs. 66.1% in control arm). Grade ≥ 3 TRAEs were reported in 14.7% of patients (vs. 3.4% in placebo group), with one treatment-related fatality (0.2%) attributed to severe myositis. IrAEs occurred in 37.3% of pembrolizumab recipients. Endocrine disorders were remarkably prominent, occurring in 23.4% of patients: hypothyroidism developed in 14.3% of patients and hyperthyroidism in 10.2%, though the vast majority were low-grade (grade 1 or 2). Crucially, severe permanent endocrinopathies occurred, including grade 3–4 type 1 diabetes mellitus (1.0%) and hypophysitis or hypopituitarism (0.6%). Other non-endocrine toxicities included any-grade pneumonitis (3.3%) and grade 3–4 colitis (2.0%). Adverse events led to treatment cessation in 13.8% of patients starting pembrolizumab, with 13.0% considered directly related to the drug regimen by the investigators [34].

Based on these data and on those obtained in the metastatic setting, we can conclude that there are no significant differences in the safety profile between the two approved anti-PD-1 agents and that there are probably no defined or relevant toxicity criteria to prefer one of them when approaching patients.

The COMBI-AD study safety analysis revealed that any-grade adverse events affected 97% of patients in the combination therapy cohort, and grade 3–4 toxicities occurred in 41%. Pyrexia was the absolute hallmark toxicity, occurring in 63% of patients (with a 5% grade 3–4) and prompting drug discontinuation across the standard 12-month timeline. Other frequent toxicities included fatigue (47%), nausea (40%), chills (37%), diarrhea (33%), arthalgia (28%), vomiting (28%), and headache (39%). Moreover, even if not frequent and almost reversible, targeted therapy can also impact on cardiac function, resulting rhythm or pump dysfunction. In addition to that, surveillance over the long-term follow-up revealed a higher incidence of secondary or primary cancers with dabrafenib plus trametinib compared to placebo (3.98 vs. 2.61 events per 100 patient-years). The most common manifestations were skin cancers, such as basal-cell carcinoma (5%) and new primary malignant melanomas (3%), heavily driven by high skin surveillance. Due to cumulative systemic side effects, 26% of patients in the combination-therapy group prematurely discontinued the clinical trial regimen. Adverse events leading to dose interruption or dose reduction occurred in 66% and 38% of subjects.

Recognizing that both dabrafenib and trametinib synergize to drive pyrexia syndrome (fever, chills, rigors, and flu-like symptoms), an adapted adverse event management algorithm was validated in the phase IIIb COMBI-APlus trial. This protocol mandates that both drugs are simultaneously held at the earliest sign of fever (≥38 °C) or prodromal symptoms and safely restarted at the same dose only after the patient is entirely symptom-free for ≥24 h. COMBI-APlus successfully achieved its primary endpoint, reducing the severe composite pyrexia rate (grade 3–4 fever, hospitalization, or permanent drug cessation due to pyrexia) from a historical baseline of 20.0% in COMBI-AD down to just 8.0%. Discontinuation due to pyrexia dropped sharply to 2.4%, and total drug discontinuations due to any adverse event fell to 14.7%. The prospective German registry study COMBI-EU translated these findings into real-world clinical care across 225 analyzed patients. The study underscored that high-level, algorithmic toxicity management (utilizing dose adjustments or strict reintroduction rules) significantly optimized treatment adherence. Real-world grade 1–2 toxicities like increased liver enzymes (36.4%), fever (30.2%), and fatigue (27.1%) were heavily reported, but total real-world drug discontinuations due to treatment-related toxicities were successfully capped at 16.4%.

As highlighted by evidence in advanced disease, the toxicity profile of targeted therapy is characteristically acute, systemic, and reversible, carrying minimal to no risk of chronic organ damage post-cessation [57].

On the other side, anti-PD-1 immunotherapy real-world monitoring has unmasked a distinct clinical challenge: the development of chronic immune-related adverse events that persist long after treatment termination. Multicenter cohort tracking across the United States and Australia reveals that 43.2% of patients treated with adjuvant anti-PD-1 therapy develop chronic irAEs (defined as toxicities persisting for 12 weeks or longer after therapy cessation). Although the vast majority of these events (96.4%) are mild to moderate in nature (grade 1 or 2), they exhibit remarkable longevity, with 85.6% remaining entirely unresolved at last follow-up. There is a profound divergence in chronicity based on the affected organ class. IrAEs targeting non-visceral structures are exceptionally prone to becoming chronic permanent fixtures. About 83.0% of acute endocrine cases transform into chronic conditions, with hypothyroidism (14.0% of the full cohort) and adrenal insufficiency (3.1%) showing a 100% persistence rate at final clinical contact. Nearly half (48.9%) of joint toxicities persist chronically, demonstrating 100% persistence through extended follow-up. In addition, salivary gland dysfunction (52.9% chronic) and ocular toxicities (62.5% chronic) carry high long-term persistence rates. In stark contrast, acute toxicities affecting larger visceral organs demonstrate rapid resolution and lower chronicity rates: only 13.6% of colitis events become chronic, and the majority of those eventually resolve over time. Chronicity is entirely uncoupled from traditional patient baselines: age, gender, the precise timing of initial acute onset, and the requirement for systemic steroids do not correlate with whether an irAE will become permanent. Strikingly, roughly 49.1% of chronic irAE cases are fully symptomatic, and 32.9% continue to require chronic steroid or hormonal replacement intervention. Despite their low grade, these persistent conditions highlight that a large subset of adjuvant-cured patients undergo permanent physiological alterations [58].

Data suggests a significant divergence in terms adjuvant melanoma treatments. While adjuvant targeted therapy is characterized by intense, but largely reversible acute symptoms, anti-PD-1 immunotherapy carries a high propensity (43.2%) for chronic toxicities that can persist indefinitely (such as inflammatory arthritis or permanent hormone deficiencies). This underscores the necessity of an individualized approach that integrates the patient’s age, baseline health, and tolerance for potential lifelong medical management, prioritizing the patient’s long-term quality of life alongside traditional oncological endpoints necessitates.

### 5.4. The “Second Adjuvant” Option

In melanoma treatment, “second adjuvant” therapy refers to a second course of adjuvant systemic treatment given to patients who completed or progressed on an initial upfront adjuvant therapy (such as standard anti-PD-1 immunotherapy), developed a locoregional or resectable metastatic recurrence, and subsequently underwent definitive local therapy (like surgical R0 resection) to become disease-free again.

Because landmark randomized controlled trials traditionally focused on systemic treatment-naïve populations, robust prospective data in the recurrent setting remain limited. However, a combination of recent clinical trial updates and multicenter retrospective registry studies provides at least general evidence on efficacy, safety, and resistance patterns.

Starting from Keynote-054, rechallenge with anti-PD-1 monotherapy showed limited efficacy in this setting: among patients initially treated with pembrolizumab who relapsed >6 months after completing therapy and were rechallenged with pembrolizumab, the objective response rate (ORR) was only 15%, with a brief median progression-free survival (PFS) of 4.1 months.

Owen et al. underscored the importance of the time of recurrence for the clinical success of a second adjuvant course: for patients who experience a recurrence on or until 1 month post-cessation adjuvant anti-PD-1, continuing or retreatment with PD-1 monotherapy yields predictably zero responses (0%) and a median PFS of just 2.3 months. Conversely, patients who recur later than 1 month post-cessation of anti-PD1 exhibit a 40% response rate to anti-PD-1 retreatment.

Based on these data, we can conclude that while anti-PD-1 retreatment has clinical utility for late recurrences well after treatment cessation, it should be entirely avoided in patients progressing while actively on therapy. Indeed, true primary or early secondary resistance mechanisms persist if a patient relapses while on treatment.

For patients with BRAF V600-mutated melanoma who relapse during or after upfront anti-PD-1 immunotherapy, switching to targeted therapy (BRAF/MEK inhibitors, such as dabrafenib + trametinib) seem to yield the highest clinical rescue rates.

Taylor et al. demonstrated that patients receiving a second adjuvant course of BRAF/MEK inhibitors following PD-1 failure experienced a vastly superior median recurrence-free survival (RFS) compared to those who did not receive second adjuvant therapy (30.8 months vs. 4 months; HR 0.35) [59].

Moreover, ADOREG Skin Cancer Registry real-world data confirmed that second adjuvant targeted therapy drastically improved second-line recurrence-free survival (RFS2) over a second round of checkpoint inhibitors (41 months vs. 6 months) [60].

Conversely, patients who fail upfront adjuvant BRAF/MEK inhibitors and are switched to a second adjuvant course of anti-PD-1 monotherapy perform poorly, frequently exhibiting rapid secondary resistance [61].

However, while second adjuvant targeted therapy or combination rescue regimens delay early recurrence and extend RFS2, mature data show no significant difference in overall survival (OS) when compared to observation or alternative delayed treatments [60]. This aligns with the Keynote-054 observation that post-relapse metastatic salvage therapies remain highly effective at long-term stage IV control.

Besides systemic therapies, for patients who experience a locoregional nodal or in-transit recurrence during or after anti-PD-1 immunotherapy, adding adjuvant radiotherapy (RT) to the local field after surgical resection acts as an effective secondary regional preventative measure. Data from a multicenter study tracking these patients showed that adjuvant RT at first recurrence drastically reduces the rate of a second locoregional relapse (8% vs. 36% in the non-RT group) and significantly improves locoregional recurrence-free survival (lr-RFS2) [62]. This is heavily supported by Owen et al., who noted that among patients experiencing a resectable locoregional relapse, surgical resection alone was followed by a high rate of subsequent relapse (56% overall, with 38% progressing to distant disease). This high failure rate reinforces the requirement for aggressive secondary regional strategies (like adjuvant RT) or secondary systemic interventions.

### 5.5. Management of Post-Recurrence

Dropping immunotherapy upon relapse and starting targeted inhibitors represents a highly effective salvage pathway for BRAF-mutant patients. Real-world validation from Italian academic institutions proves that tumor escape from checkpoint inhibitors does not compromise downstream sensitivity to MAPK pathway suppression. Consequently, patients facing adjuvant anti-PD-1 failure remain highly responsive to first-line targeted regimens, achieving excellent objective response rates and durable disease control upon developing advanced stage IV disease [63].

Frontline adjuvant targeted therapy does not seem to exhaust the patient’s downstream immunologic reserve, as shown by Bhave et al. demonstrating that resected individuals who relapse after completing their targeted course retain full sensitivity to subsequent anti-PD-1 checkpoint blockades. Advanced-stage response rates to first-line anti-PD-1 monotherapy or dual checkpoint therapies like ipilimumab and nivolumab remain robust in these cohorts, closely tracking historical benchmarks established in completely treatment-naive metastatic settings. This indicates that the finite, 12-month duration of adjuvant targeted therapy successfully circumvents the deep, multi-clonal resistance profiles typically seen when these small-molecule inhibitors are administered continuously until progression in advanced settings [62]. A stark clinical contrast emerges, however, for patients experiencing rapid disease progression while actively on targeted agents, as they display a highly aggressive cross-resistance phenotype that compromises single-agent immunotherapies. As suggested, on-treatment tumor adaptations construct a hostile, glucose-starved microenvironment where severe nutrient competition and massive lactate accumulation physically blunt effector T-cell and NK-cell activity. Biopsies matching this rapid targeted failure profile reveal a stark absence of active T cells and downregulated tumor antigens alongside heavy expression of exhaustion checkpoints, specifically TIM-3 and PD-1 [64]. Standard single-agent monotherapy rescue holds exceptionally low efficacy in these immediate, on-treatment resistance scenarios, and it was noted that these cases require treatment class switching or dual-checkpoint escalation to bypass multi-agent biological resistance mechanisms [59,65]. This clinical landscape is meticulously contextualized across post-progression trial analyses in a post hoc analysis starting from CheckMate 238 data, which outlines that approximately two-thirds of immunotherapy relapses manifest early, typically while patients are still actively taking the adjuvant drug. It was clarified that these early progressors derive minimal benefit from anti-PD-1 rechallenges, unlike late progressors who relapse more than six months off-drug. Instead, early progressors achieve superior systemic rescue by shifting directly to combined dual immunotherapy or class-switching to targeted MAPK-pathway inhibitors [66].

### 5.6. The Choice in BRAF-Mutated Melanoma

As seen previously, the presence or absence of the *BRAF* V600 mutation and consequently the possibility of prescribing in the adjuvant setting dabrafenib + trametinib rather than anti-PD-1 concerns only stage III, this doublet not being approved for stages II or IV. Most clinical considerations on this topic stem from the analysis of the Keynote-054 and the Combi-AD trials. Table 4 provides a comparison of these two regimens in this setting, taking into account efficacy, safety, and patient and disease management concerns.

These studies are highly comparable due to their similar inclusion criteria: both utilized randomization against a placebo, enrolled homogeneous stage III populations (excluding stage IIIA with <1 mm sentinel node burden), and required complete lymphadenectomy. Conversely, applying results from CheckMate 238 is more complex, as that trial utilized an active comparator (high-dose ipilimumab), excluded all stage IIIA disease, and included resected stage IV patients. Notably, *BRAF*-mutant population was well represented across both anti-PD-1 trials, comprising 41% of the CheckMate 238 cohort and 50% of Keynote-054.

Both adjuvant anti-PD-1 studies demonstrated that efficacy was generally maintained across all subgroups, including *BRAF* mutational status. Specifically, CheckMate 238 reported a 3-year recurrence-free survival (RFS) of 56% for *BRAF*-mutant subjects (HR 0.79; 95% CI 0.59–1.06) compared with 60% in *BRAF* wild-type patients (HR 0.60; 95% CI 0.45–0.80) [67]. At a 5-year follow-up, nivolumab continued to show superior RFS and DMFS rates over ipilimumab, both in *BRAF*-mutant patients (50% vs. 42 and 61% for RFS and 61% vs. 56% for DMFS, respectively) and wild-type patients (47% vs. 36% for RFS and 56 vs. 51% for DMFS, respectively). Initial overall survival (OS) data from this trial suggested a more pronounced benefit for nivolumab in the wild-type cohort, though OS did not significantly differ between treatment arms given the current data maturity (75 and 73% in the two groups) [68].

In the Keynote-054 trial, pembrolizumab showed robust efficacy in both *BRAF*-mutant and wild-type tumors, with 3-year RFS substantially improved over placebo (62.0% vs. 37.1%; HR 0.51 and 61.8% vs. 46.5%; HR 0.66, respectively). The DMFS outcomes mirrored these RFS findings, demonstrating a protective effect against distant spread across both molecular subgroups (67% vs. 44% HR 0.53 for *BRAF* mutant; 65% vs. 54% HR 0.73 for wild-type). While OS remains a pending secondary endpoint awaiting full maturity, a cross-trial comparison of 3-year data reveals no striking differences between anti-PD-1 agents and the RFS achieved with targeted therapy in the COMBI-AD trial.

Comparing the 3-years efficacy data of anti-PD-1 in mutated patients with the 3-year RFS obtained in Combi-AD with targeted therapy, we can notice that at this follow-up there are no clear differences between the two therapeutic approaches in setting, even if there are obvious limitations due to the indirect comparison among trials.

It is not possible today to define what the first-choice adjuvant therapy is in patients with stage III resected *BRAF*-mutated melanoma. Among factors clinical oncologists should take into account in these cases, we would mention the following.

➢ Specific contraindications of the two classes of treatment. For example, in the presence of QTc interval prolongation, retinal vein occlusion history or thrombotic diathesis, immunotherapy should be preferred. On the other hand, targeted therapy must be considered as treatment of choice, if feasible, in cases of serious autoimmune disease, solid organ transplantation, immunosuppression or immune-depressive conditions.
➢ Clinical and social frailty, the presence of a caregiver and the compliance in adverse events reporting. These factors are mostly (but not exclusively) important for old patients: as largely discussed before, targeted therapy and immunotherapy present different safety profiles. If treatment with dabrafenib + trametinib is associated with a greater overall risk of G3–4 AEs and permanent suspension of therapy due to toxicity, serious side effects caused by immunotherapy are more difficult to manage, requiring the use of high-dose corticosteroids or other immunosuppressants, are often chronic and can, if not rapidly and correctly managed, put the patient’s life at risk. For these reasons the prompt recognition of toxicities could impact on the possibility of effectively treat them without life-treating sequelae.
➢ Compliance with oral or intravenous rout of administration.
➢ Patient’s lifestyle (highly irregular sleeping habits or mealtimes).
➢ Comorbidities that can make the management of adverse events complex. For example, using steroids to treat immune-related toxicities may exacerbate hyperglycemia in diabetic patients and potentially compromising cardiovascular function in those with preexisting heart conditions.
➢ Potential impact on reproductive system (fertility, sexual dysfunction).
➢ Concomitant therapies that could interfere with the activity and toxicity of the adjuvant agents (i.e., immunosuppressant for immunocheckpoint inhibitors or CYP3 A4-CYP2 C8 strong inducers or inhibitors in the case of targeted therapy). Moreover, the impact of adjuvant agents on activity and toxicity of home medications should be considered.
➢ Disease substage could be evaluated as an element for the personalization of adjuvant treatment.

Stage IIIA: as seen before, these patients generally have a good prognosis, and data about this substage are scarce. For these reasons, in *BRAF*-mutated patients in stage IIIA, particularly in cases of limited involvement in the sentinel lymph node, targeted therapy could be preferred to immunotherapy to avoid long-term unnecessary toxicities.

Stage IIID: this substage was also poorly represented in clinical trials, but due to the unfavorable prognosis, numerous events have been observed among subjects treated with immunotherapy: almost 50% experienced disease recurrence at the first tumor assessment performed at 3 months. For patients that underwent anti-PD-1, the 3-year RFS was lower than for those that underwent dabrafenib + trametinib (HR 0.68 [99% CI of 0.24–1.91] vs. 0.34 [95% CI 0.14–0.79]), so targeted therapy should probably be preferred to avoid the risk of early relapses, even if long-term data are needed.

➢ Type of *BRAF* mutation: targeted therapy was tested only in BRAF V600 E/K melanomas, and while RFS benefit was confirmed for both type of mutation, the positive trend on OS was suggested only for BRAF V600 K.
➢ Estimated risk of immediate/early relapse (during therapy or early after discontinuation). Retrospective, real-world data from the international ADOREG registry alongside the German Dermatologic Cooperative Oncology Group (DeCOG) cohort reveal that initial therapeutic choices drastically alter early disease-free intervals. In particular, patients assigned to adjuvant anti-PD-1 are highly vulnerable to rapid, “on-treatment” progression. Conversely, those receiving adjuvant targeted therapy typically enjoy early protection, only to experience a rebound of disease relapse “off-treatment” heavily concentrated within the first 6 months following their 12-month treatment completion. This disparity is further validated by multicenter cohort data in another multicenter retrospective cohort study involving 15 melanoma centers: frontline targeted agents significantly cut immediate recurrence risk with respect to adjuvant immunotherapy. Ultimately, this clinical divergence underscores the distinct, rapid mechanism of early MAPK pathway inhibition compared to the delayed immune-priming kinetics of standard checkpoint inhibition in real-world practice [60,63,69,70].
➢ Previous therapy received in the adjuvant setting (see “The “second adjuvant” option” paragraph).
➢ Previous therapy received in the neoadjuvant setting and response to the administered regimen (see “The new medical management of stage III melanoma: the neoadjuvant setting” option paragraph).

### 5.7. The New Surgical Management of Stage III Melanoma

The shift in the management of resected stage III melanoma represents a major evolution in surgical and medical oncology, having influenced the design and generalizability of adjuvant therapy trials across time.

Until 2017, the standard of care for patients with a positive sentinel lymph node (SLN) was complete lymphadenectomy (CLND). This was a mandatory inclusion criterion for landmark adjuvant trials such as CheckMate 238 and Keynote-054.

The MSLT-II and DeCOG-SLT trials changed this paradigm. MSLT-II demonstrated that while immediate CLND provided better regional nodal control, it did not improve melanoma-specific survival compared to active surveillance with ultrasound in patients with SLN micrometastases. DeCOG-SLT similarly found no survival benefit (RFS, DMFS, or OS) for patients undergoing CLND versus those managed with observation after a positive sentinel node biopsy [71,72].

During recruitment for CheckMate-915 (from April 2017 to June 2018), clinical practice was transitioning away from routine CLND. As such, in contrast with previous adjuvant therapy trials that included only patients that had performed CLND in cases of positivity of sentinel-node biopsy, for CheckMate-915 this was not among inclusion criteria. Consequently, while the CheckMate-915 data are directly translatable to clinical practice excluding an improvement of RFS for the doublet, but reaffirming nivolumab monotherapy as a reliable standard of care in a modern surgical context [39].

Conversely, the exclusion of CLND in modern practice raises questions about whether the efficacy established in older trials (where CLND was mandatory) remains consistent in patients managed with surveillance only confirming previous cost-effectiveness assessments. This should be confirmed prospectively in populations of patients who have not undergone complete lymphadenectomy.

While investigators speculate that the relative benefit of adjuvant therapy remains stable, recent real-world evaluations have begun to confirm that active surveillance combined with anti-PD-1 therapy yields outcomes comparable to the high-risk cohorts seen in the original randomized trials.

Different trials address this issue, suggesting that the different surgical management of these patients did not significantly impact treatment effectiveness for anti-PD-1 or targeted adjuvant therapy [72,73,74,75]. As such, we can conclude that the current evidence supports the use of adjuvant treatment regardless of whether a patient has undergone CLND. However, for the specific population of sentinel node-positive patients who defer CLND, continued surveillance with high-quality nodal ultrasound is essential to identify regional failures early enough for definitive therapeutic dissection if recurrence occurs.

### 5.8. The New Medical Management of Stage III Melanoma: The Neoadjuvant Setting

In the last few years, the management of resectable macroscopic stage III melanoma has fundamentally shifted from upfront surgery followed by empirical adjuvant therapy to a proactive, biologically driven neoadjuvant approach. Immunologically, administering checkpoint inhibitors while the macro-metastatic tumor bulk and its microenvironment remain intact exposes the immune system to a broader diversity of tumor-associated neoantigens. This rich antigenic landscape drives a more robust, systemic clonal expansion of tumor-specific T cells capable of eradicating distant micrometastases. Conversely, delaying systemic therapy until after a therapeutic lymph-node dissection eliminates this priming environment and leaves a depleted regional immune architecture. Beyond the immunological advantages, the neoadjuvant window offers a vital in vivo readout of tumor biology, transforming the pathological response into a dynamic biomarker to guide personalized postsurgical care.

The clinical validation of this framework evolved through two distinct research lines that systematically optimized treatment sequencing, drug dosing, and surgical management.

First, the randomized phase II SWOG S1801 trial established the definitive proof of concept for timing, showing that splitting a standard pembrolizumab backbone into a perioperative sequence yielded superior 2-year event-free survival compared to an identical adjuvant-only regimen (72% vs. 42%). Concurrently, the OpACIN and OpACIN-neo trials sought the ideal systemic combination, ultimately identifying a therapeutic “sweet spot” by flipping the standard dosing to low-dose ipilimumab (1 mg/kg) plus standard-dose nivolumab (3 mg/kg). This modification preserved a major pathological response (MPR) rate near 60% while successfully halving severe immune-related toxicities. Finally, the PRADO extension trial operationalized this high efficacy into surgical practice: by confirming that patients achieving an MPR in a single “index” lymph node could safely omit both complete nodal clearance and adjuvant therapy, PRADO provided the definitive blueprint for response-adapted de-escalation.

Together, these milestones served as the direct methodological launching pad for the definitive phase III validation: the NADINA trial. This practice-changing trial directly compared two cycles of neoadjuvant ipilimumab plus nivolumab followed by response-driven surgery and adjuvant therapy against upfront surgery followed by one year of adjuvant nivolumab. The neoadjuvant arm demonstrated a striking 68% reduction in the risk of progression, recurrence, or death, with a 12-month EFS of 83.7% vs. 57.2%. Nearly 60% of neoadjuvant patients achieved an MPR, allowing them to completely omit adjuvant therapy and conclude their entire systemic regimen in just six weeks.

It was precisely this historical momentum that contextualized the phase III NADINA trial, a study that forced a definitive re-evaluation of macroscopic stage III melanoma care. Investigators compared a truncated neoadjuvant regimen—consisting of just two cycles of combination ipilimumab and nivolumab before surgery—against classical immediate resection followed by a full year of adjuvant anti-PD-1 monotherapy. The resulting data fundamentally rewrote the prognosis for this population: the neoadjuvant arm demonstrated a profound 68% reduction in the risk of clinical events, translating to a 12-month EFS advantage of 83.7% over 57.2%. Beyond the survival benefit, the trial operationalized a dramatic reduction in treatment burden: an MPR rate of nearly 60% allowed the majority of patients to entirely forgo adjuvant intervention, compressing a historically year-long therapeutic ordeal into a highly efficient, six-week frontline protocol.

Indeed, the response-adapted framework of this study expects that responders (MPR) bypass adjuvant therapy entirely, significantly improving quality of life by avoiding prolonged drug toxicities. Conversely, pathological non-responders (>50% viable tumor) are identified early as refractory to standard immunotherapy and could pivot to systemic management starting dabrafenib + trametinib in the presence of *BRAF* V600 E/K mutation.

Data from the NADINA trial revealed that neoadjuvant dual checkpoint inhibition is equally effective regardless of mutational status, yielding virtually identical 12-month EFS rates in both BRAF-mutant (83.5%) and BRAF wild-type (83.9%) arms. Consequently, a baseline BRAF mutation should not deter the use of upfront dual immunotherapy. Instead, the BRAF mutation serves as a precise, personalized safety net at the time of surgery. If a BRAF-mutant patient exhibits pathological non-response after neoadjuvant immunotherapy, clinicians have definitive proof of checkpoint resistance. Rather than continuing futile anti-PD-1 therapy, they can immediately exploit the tumor’s genetic vulnerability by switching to adjuvant targeted therapy with BRAF/MEK inhibitors. This biology-driven sequence reserves targeted agents as a highly effective rescue mechanism for the exact subset of patients who require an alternative mechanism of action.

### 5.9. Optimal Duration of Treatment

Since 2018, 12 months of adjuvant PD-1 inhibition or targeted therapy has become the standard of care for high-risk melanoma. This duration is established by landmark trials such as CheckMate 238, Keynote-054, and COMBI-AD [31,34,46]. More recently, the NADINA and SWOG S1801 [76,77] studies have highlighted the superior efficacy of neoadjuvant (presurgical) treatment compared to the standard administration of anti-PD-1 monotherapy in the postsurgical phase alone.

Despite these advancements, the 12-month timeframe remains an arbitrary benchmark established by registered trials rather than a scientifically optimized duration. Emerging evidence raised the hypothesis that a full year of anti-PD-1 treatment could be unnecessary; for instance, German research has indicated that six months of treatment may be as effective as twelve [78]. Furthermore, in neoadjuvant settings, many patients achieve a major pathological response (MPR) after only a few doses [76,77]. From a biological and immunological perspective, it is well established that—unlike chemotherapy—immunotherapy can generate long-lasting memory T cells that continue to provide protection long after the final dose.

Drawing a parallel to other malignancies, such as colorectal and breast cancer, large non-inferiority trials have successfully reduced treatment durations (e.g., from six months down to three) without compromising outcomes [78,79,80]. Investigating a reduced duration for anti-PD-1 administration is therefore critical, as it would likely improve patient quality of life, reduce healthcare costs, and mitigate the risk of high-grade or chronic toxicities.

Against this background, the GrandSLAM trial—a prospective, phase III, randomized, controlled international multicenter non-inferiority study—is currently enrolling participants. Its primary objective is to determine whether a shorter (6-month) duration of (neo)adjuvant immunocheckpoint inhibition is as effective as the standard (12-month) duration regarding distant metastasis-free survival (DMFS) and relapse-free survival (RFS) in patients with resected stage IIB–C, III, or IV cutaneous melanoma. In cases where patients receive neoadjuvant treatment, the adjuvant phase is shortened to two months. Notably, subjects who achieve an MPR following preoperative administration are excluded from the study [81].

Conversely, the same arbitrariness applies to the one-year duration of targeted therapy. Considering the distinct mechanisms of action—which often discourage therapy discontinuation in the metastatic setting—it could be suggested that a longer duration of dual BRAF–MEK inhibition in the adjuvant setting should be investigated to potentially enhance treatment performance.

In conclusion, while 12 months remains the global norm, there is a critical need for randomized clinical trials to optimize these therapeutic windows.

### 5.10. Role of Adjuvants in Oligometastatic Disease

Although the CheckMate 238 and SWOG S1404 trials evaluated adjuvant therapy in resected stage IV melanoma—specifically comparing anti-PD-1 to ipilimumab in the former, and pembrolizumab to ipilimumab or interferon alpha-2 b in the latter—the most robust data for this population currently stem from the IMMUNED trial.

Evidence from IMMUNED demonstrates that compared to placebo, both anti-PD-1 monotherapy and the nivolumab-plus-ipilimumab combination significantly reduce the risk of recurrence. At a median follow-up of 49.2 months, the 4-year recurrence-free survival (RFS) rates were 64.2% for the combination group, 31.4% for nivolumab monotherapy, and 15.0% for the placebo group. The hazard ratios (HRs) for recurrence were 0.25 for the combination (*p* < 0.0001) and 0.60 for nivolumab monotherapy (*p* 0.024) versus placebo. Furthermore, an exploratory analysis indicated the superiority of the combination over monotherapy (HR 0.41, *p* 0.001), although the trial was not powered for a direct head-to-head comparison between the two active arms [45,46].

Regarding overall survival (OS), the median has not yet been reached in any treatment group. However, 4-year OS rates were 83.8% for the combination, 72.6% for nivolumab alone, and 63.1% for placebo. Nivolumab plus ipilimumab demonstrated a significant OS benefit compared to placebo (HR 0.41, *p* 0.040), whereas nivolumab monotherapy did not show a statistically significant improvement in OS over placebo (HR 0.75, *p* 0.44) [45,46].

In light of these findings, it is important to note that while the nivolumab-plus-ipilimumab combination is included in the NCCN guidelines, it has not yet been formally registered by regulatory agencies for adjuvant use. Considering the OS data from pivotal immunotherapy and targeted therapy trials in the first line metastatic setting—particularly in patients with low tumor burden—along with recent real-world evidence suggesting that upfront anti-PD-1 therapy is at least equivalent to upfront surgery followed by adjuvant therapy, it is argued that medical therapy should be prioritized. This perspective is further supported by recent neoadjuvant evidence and current ESMO recommendations, which suggest that upfront systemic therapy is the preferred approach for oligometastatic disease.

### 5.11. Overall Survival Impact

At present, no direct evidence from phase III clinical trials demonstrates a statistically significant overall survival (OS) benefit for currently used adjuvant therapies across any stage of melanoma. In particular, the final analysis for adjuvant dabrafenib plus trametinib in stage III disease—conducted after a 10-year follow-up—confirmed superior recurrence-free survival (RFS) and distant metastasis-free survival (DMFS) compared to placebo. While the analysis showed that the risk of death was 20% lower, this benefit did not reach statistical significance (*p* 0.06) [48]. On the other hand, nivolumab has not been associated with a significant OS improvement over placebo or active comparators in trials like CheckMate 238 or IMMUNED [33,44]. Furthermore, the final OS analysis from the pivotal Keynote-054 trial has been delayed until late 2026 but the crossover design could make this assessment more difficult. Retrospective real-world studies from Sweden and the United States have also yielded conflicting results regarding survival advantages in routine clinical practice, further complicating the assessment of long-term societal benefits. As such, we can state that while modern adjuvant therapies have shown strong and durable RFS/DMFS benefit, OS remains difficult to prove because of crossover design, existence of effective salvage therapies, power of trials, and long post-recurrence survival. However, local regulatory agencies prioritize OS as a critical benchmark: consequently, European reimbursement policies vary widely [82]. For all these reasons, we can conclude that, even if adjuvant therapy is today considered a cornerstone in the modern treatment of melanoma, its definitive role and other specific aspects require further clarification and investigation.

## 6. Conclusions

Over the last decade, the management of radically resected stage III and IV cutaneous melanoma has completely evolved: from the past toxic, modest benefits of interferon-alpha to modern and sophisticated approaches based on immunotherapies and targeted agents. As this review demonstrates, anti-PD-1 checkpoint inhibitors (pembrolizumab and nivolumab) alongside dual MAPK pathway inhibitors (dabrafenib plus trametinib) have fundamentally rewritten postoperative care, offering high-risk patients a highly durable shield against both regional and distant recurrence.

However, these clinical milestones have also exposed several gray areas where clear guidelines fall short and treatment must be personalized. Key challenges remain, particularly finding the right path for stage IIIA patients, setting the sequence for rescue therapies in stage IV disease, navigating choices in BRAF-mutant cohorts, and addressing the ongoing lack of clear overall survival (OS) benefits in modern clinical trials. Modern oncology has successfully broken down the historical 90% relapse rate. Now, the future of high-risk melanoma care hinges on smart therapeutic de-escalation, tight algorithmic side-effect management, and response-driven perioperative schedules. Moving away from a rigid, 12-month treatment template toward flexible, biology-led interventions is crucial if we are to safeguard the patient’s long-term quality of life while still achieving essential oncological outcomes.

## Figures and Tables

**Table 1:** Standard-of-care systemic adjuvant therapy for stage III and IV cutaneous melanoma according to FDA and EMA approvals in 2026.

Therapeutic Agent(s)	Stage AJCC 8th Edition	Trial Supporting Evidence	Molecular Requirements	Regulatory Approval (Present and First)		Guidelines Recommendation (2026)	
				FDA	EMA	NCCN	ESMO
Nivolumab 3 mg/kg Q2 W	Stage III	Checkmate-238	NO	Stage III (2026)		Stage III *	
				In presence of nodal or metastatic involvement (2017–2018)			
	Stage IV	Checkmate-238	NO	Stage IV		Stage IV	
Pembrolizumab 200 mg Q3 W	Stage III	Keynote-054	NO	Stage III (2026)		Stage III *	
				In presence of lymph-node involvement (2019)			
Dabrafenib 150 mg BID + Trametinib 2 mg QD	Stage III	COMBI-AD	BRAF V600-mutant	In presence of lymph-node involvement BRAF V600 E-K	Stage III BRAF V600 mutant	Stage III * BRAF V600 mutant	stage III * BRAF V600 E-mutant ^§^
Ipilimumab 10 mg/kg Q3 W	Stage III	E1609 EORTC 1807	NO	In presence of pathological involvement of regional lymph nodes (≥1 mm) who have undergone complete resection	NO	Only in certain circumstances ^^^	NO

* To consider after radical surgery following recurrence during or after exposure to anti-PD1. ^§^ According to the ESMO, adjuvant dabrafenib + trametinib is a standard-of-care adjuvant treatment option for BRAF V600 E-mutated stage III melanoma, while targeted therapy should not be offered to patients with BRAF V600 K-mutated melanoma in light of the potential detrimental effect on OS. ^^^ For AJCC 8th edition stage IIIA and <1 mm SLNB tumor burden, in the absence of prospective validation of the benefit of adjuvant therapy in this patient population, adjuvant therapy should not be considered as the standard treatment.

**Table 2:** Pivotal trials on adjuvant immunocheckpoint inhibitors and BRAF-targeted therapy in completely resected stage III–IV cutaneous melanoma.

Trial	Target Population	Enrolled Patients	Study Design	Primary Endpoint	Median FU at Latest Update	RFS-DMFS-OS	Toxicity ≥ G3
** EORTC 18071 **	AJCC 7th edition stage III after completion lymphadenectomy Key exclusion criteria: - in-transit only metastases - stage IIIA with a nodal tumor burden less than 1 mm	915	Ipilimumab 10 mg/kg every 3 weeks × 4 → every 3 months for up to 3 years (ipi10) vs. placebo	RFS	6.9 y	7 y RFS: 39.2% vs. 30.9% (HR 0.75; *p* < 0.001) 7 y DMFS 44.5% vs. 36.9% (HR 0.76; *p* = 0.002) 7 y OS: 60.0% vs. 51.3% (HR 0.73; *p* = 0.002)	54.1%
** E1609 **	AJCC 7th edition stage IIIB, IIIC after completion of lymphadenectomy or resected M1 b	1670	Ipilimumab 3 mg/kg * vs. ipilimumab 10 mg/kg * vs. standard intravenous HDI * Both administered every 3 weeks × 4 → every 12 weeks for up to 60 weeks	RFS and OS * * ipi 3 vs. HDI and ipi10 vs. HDI	57.4 months	ipi 3 vs. HDI 5 y OS 72% vs. 67% (HR 0.78; *p* = 0.044) mRFS 4.5 vs. 2.5 y (HR 0.85 *p* = 0.065)	HDI 78.8% ipi3 38.6% ipi10 57.9%
						ipi 10 vs. HDI 5 y OS 70% vs. 65% (HR 0.88, NS) mRFS 3.9 vs. 2.4 y (HR 0.84, NS)	
** CheckMate 238 **	AJCC 7th edition stage IIIB, IIIC After completion of lymphadenectomy or resected stage IV	906	Nivolumab 3 mg/kg every 2 weeks for up to one year vs. ipilimumab 10 mg/kg every 3 weeks × 4 → every 12 weeks for up to one year	RFS	9 y	9 y RFS 44% vs. 37% med 61.1 vs. 24.2 months (HR 0.76) 9 y DMFS 54% vs. 48% med 9 y vs. 83.8 months (HR 0.81) 9 y OS 69 vs. 65% (HR 0.88, NS)	nivo 15% ipi 46%
** Keynote-054 **	AJCC 7th edition stage IIIA, IIIB, IIIC After completion of lymphadenectomy Key exclusion criteria: - in-transit only metastases - stage IIIA with a nodal tumor burden less than 1 mm	1019	Pembrolizumab 200 mg every 3 weeks for up to one year vs. placebo	RFS	7 y	7 y RFS 50% vs. 36% HR 0.63	14.7%
** SWOG S1404 **	AJCC 7th edition stage IIIA (N2 a), IIIB, IIIC After completion lymphadenectomy or resected stage IV Key exclusion criteria: - stage IIIA allowed if N2 a - acral/mucosal melanoma included	1301	Pembrolizumab 200 mg every 3 weeks for up to one year vs. SOC (ipilimumab 10 mg/kg or HDI)	RFS and OS in the subgroup with PD-L1-positive tumors	47.5 months	RFS HR 0.77 (intent-to-treat population) RFS HR 0.69 (PD-L1-positive population) OS HR 0.82 (*p* 0.15) (intent-to-treat population) OS HR 0.84 (*p* 0.29) (PD-L1-positive population)	pembro 19.5% IFN 71.2% ipi 49.2%
** CheckMate-915 **	AJCC 8th edition resected Stage IIIB–D (complete lymphadenectomy not mandatory) or IV	1883	Ipilimumab 1 mg/kg every 6 weeks plus nivolumab 240 mg every 2 weeks vs. nivolumab 480 mg every 4 weeks for up to one year	RFS and RFS in the subgroup with PD-L1-negative tumors	23.7 months	2-year RFS 64.6% (ipi + nivo) 63.2% (nivo) HR 0.92 (*p* 0.269) (intent-to-treat population) 2-year RFS 53.6% (ipi + nivo) 52.4% (nivo) HR 0.91 (NS) (PD-L1 negative population)	ipi-nivo 32.6% nivo 12.8%
** IMMUNED **	AJCC 8th edition NED stage IV after resection or radiation therapy	167	Ipilimumab 3 mg/kg plus nivolumab 3 mg/kg vs. nivolumab 3 mg/kg + placebo vs. double placebo for up to one year	RFS	23.7 months	4 y RFS 64.2% (ipi + nivo) 31.4% (nivo + placebo) 15% (double placebo) HR 0.25 for ipi + nivo vs. placebo (*p* < 0∙0001) HR 0.60 for nivo vs. placebo (*p* 0.024) 4-year OS 83.8% (ipi + nivo) vs. 72.6% (nivo + placebo) vs. 63.1% (double placebo) HR 0.41 for ipi + nivo vs. placebo (*p* < 0.040) HR 0.75 for nivo vs. placebo (*p* 0.44)	ipi-nivo 71% nivo 29%
** COMBI-AD **	AJCC 7th edition stage IIIA, IIIB, IIIC After completion of lymphadenectomy	870	Dabrafenib (150 mg twice daily) plus trametinib (2 mg once daily) vs. placebo for up to one year	RFS	8.3 years	RFS HR 0.52 for dabrafenib + trametinib vs. placebo	Dabrafenib + trametinib 41%

**Table 3:** Stage III restaging from AJCC 7th to 8th edition (adapted from: Grob JJ, Schadendorf D, Lorigan P, et al. Eighth American Joint Committee on Cancer (AJCC) melanoma classification: Let us reconsider stage III. *Eur. J. Cancer* 2018;91:168–170. doi:10.1016/j.ejca.2017.11.023).

Pathological Stage III Substage	AJCC 7th Edition Staging System	AJCC 8th Edition Staging System
IIIA	T1–4 a N1 a M0 T1–4 a N2 a M0	T1 a ^a^/b ^a^ N1 a ^a^ M0 T1 a ^a^/b ^a^ N2 a ^a^ M0
IIIB	T1–4 b N1 a M0 T1–4 b N2 a M0 T1–4 a N1 b M0 T1–4 a N2 b/c M0	T1 a ^a^/b ^a^–T2 a N1 b ^a^/c ^a^ M0 T1 a ^a^/b ^a^–T2 a N2 b ^a^ M0 T2 b–T3 a N1 a ^a^ M0 T2 b–T3 a N2 b ^a^ M0
IIIC	T1–4 b N1 b M0 T1–4 b N2 b/c M0 Any T N3 M0	T1 a ^a^–T3 a N2 c ^a^ M0 T1 a–T3 a N3 a ^b^/b ^b^/c ^b^ M0 T3 b–T4 a Any N ≥ N1 M0 T4 b N1 a ^a^ M0 T4 b N2 c ^a^ M0
IIID ^c^		T4 b N3 a ^b^/b ^b^/c ^b^ M0

^a^ Change in pathological definition, ^b^ new pathological categories, ^c^ new pathological stage.

**Table 4:** Comparison between anti-PD-1 and targeted therapy in the adjuvant setting. Variables are assessed over placebo and data obtained, based on indirect comparison, from the two most comparable adjuvant trials publish until 2026: MK 3475-054 (pembrolizumab vs. placebo) and COMBI-AD (dabrafenib + trametinib vs. placebo).

Variables	Pembrolizumab 200 mg Q3 W			Dabrafenib 150 mg BID + Trametinib 2 mg QD	
	FU (Median)	ITT Population vs. Placebo	BRAF-Mutant pts vs. Placebo *	FU (Median)	ITT Population vs. Placebo
** RFS **	15 months	1 y RFS 75.4% vs. 61.0% HR 0.54 (95% CI 0.42 to 0.69; *p* < 0.001)	HR 0.59 (95% CI 0.38–0.92)	2.8 years	1 y RFS 88% vs. 56% 2 y RFS 67% vs. 44% 3 y RFS 58% vs. 39% HR 0.47 (95% CI 0.39 to 0.58; *p* < 0.001)
	36.6 months	3 y RFS 63.7% vs. 44.1% HR 0.56 (95% CI 0.47 to 0.68; *p* < 0.001)	3 y RFS 62.0% vs. 37.1% HR: 0.52 (99% CI 0.37 to 0.75)		
	42.3 months	3.5 y RFS 59.8% vs. 41·4% HR 0.59 (95% CI 0.49–0.70)	NA	-	-
	6.9 years	5 y RFS 56% vs. 39% 7 y RFS 50% vs. 36% HR 0.63 (95% CI 0.53–0.74)	5 y RFS 55% vs. 35% 7 y RFS 50% vs. 33% HR 0.59 (95% CI 0.43–0.83)	58–59 months (minimum)	5 y RFS 52% vs. 36% HR 0.51 (95% CI 0.42 to 0.61)
	-	-	-	8.33 years	10-yr RFS 48% vs. 32% HR 0.52 (95% CI 0.43 to 0.63)
** DMFS **	15 months	18-months cumulative incidence of distant metastases 16.7% vs. 29.7% HR 0.53 (99% CI 0.37–0.76)	NA	2.8 years	HR 0.51 (95% CI 0.40–0.65); *p* < 0.001)
	36.6 months	3 y cumulative incidence rate of distant metastasis 22.3% vs. 37.3% HR 0.55 (95% CI 0.44 to 0.69)	NA		
	42.3 months	3.5 y DMFS 65.3% vs. 49.4% HR 0.60 (95% CI 0.49–0.73; *p* < 0.0001)	3.5 yDMFS 62.0% vs. 37.1% HR 0.52 (95% CI 0.37–0.75)	-	-
	6.9 years	5 y RFS 61% vs. 45% 7 y RFS 54% vs. 42% HR 0.64 (95% CI 0.54–0.76)	HR 0.59 (95% CI 0.43–0.83)	58–59 months (minimum)	5 y DMFS 65% vs. 54% HR 0.55 (95% CI 0.44 to 0.70)
	-	-	-	8.33 years	10 y DMFS 63% vs. 48% HR 0.56 (95% CI 0.44 to 0.71)
**Comorbidies Contrains**	Contraindicated in cases of active autoimmune diseases (e.g., ulcerative colitis or rheumatoid arthritis) or in organ transplant recipients			Contraindicated in patients with baseline cardiac dysfunction (low LVEF), severe unmanageable hypertension, a history of retinal vein occlusion (RVO), or severe chronic pyrexia syndrome	
**Toxicity Profile**					
**Class Effect AEs**	Immune-related adverse events			Pyrexia, fatigue, chills, diarrhea, skin toxicity, cardiac toxicity	
**G ≥3 AEs**	14.7%			41%	
**Discontinuation Rate**	13.8%			26%	
**Dose Interruption Rate**	23.3%			66%	
**Dose Reduction Rate**	NA			38%	
**Administration Logistics**	Requires periodic intravenous (IV) outpatient infusions (every 3–6 or 2–4 weeks) for 1 year			Entirely oral self-administration (daily pills) for 1 year	
**Compliance Concerns**	Correct ad prompt reporting of adverse events			Correct and punctual administration	
**Salvage Therapies upon Recurrence**	If a patient relapses while on adjuvant anti-PD-1, they are generally resistant, and switching to targeted therapy or dual immunotherapy has lower salvage success			If a patient relapses after completing adjuvant targeted therapy, tumor sensitivity to immunotherapy is preserved. They respond exceptionally well to first-line metastatic immunotherapy	

* Prespecified subgroup analysis performed for exploratory purposes.

## Data Availability

No new data were created.
